# Prevalence and Determinants of Pregnancy‐Induced Hypertension Among Women Attending Antenatal Care in Asella Town, Southeast Ethiopia: A Cross‐Sectional Study

**DOI:** 10.1002/hsr2.73189

**Published:** 2026-09-01

**Authors:** Birtukan Ahmed, Ayalneh Demissie, Biniam Belete, Addis Wordofa, Helen Bekele, Tesfa Gebremeskel

**Affiliations:** ^1^ Arsi Zonal Health Department Arsi Zone Health Bureau Asella Ethiopia; ^2^ Department of Public Health College of Health Sciences Arsi University Asella Ethiopia; ^3^ ASTU Student Health Center Adama Science and Technology University Adama Ethiopia; ^4^ Department of Paediatrics College of Health Sciences Arsi University Asella Ethiopia

**Keywords:** antenatal care, Asella, Ethiopia, pregnancy‐induced hypertension, prevalence, STROBE

## Abstract

**Background and Aims:**

Pregnancy‐Induced Hypertension (PIH) is a primary contributor to global maternal and perinatal mortality. This study determined the prevalence of PIH and identified its modifiable and non‐modifiable determinants among pregnant women attending antenatal care (ANC) in Asella Town, southeast Ethiopia.

**Methods:**

A facility‐based cross‐sectional study was conducted in December 2023 across three public health facilities. Systematic random sampling selected 834 pregnant women with a gestational age greater than 20 weeks. Data were collected through structured interviewer‐administered and medical chart reviews. Statistical analyses were performed using STATA Software, version 17.0 (StataCorp LLC, College Station, TX, USA). Variables with a *p*‐value less than 0.25 in bivariable analysis were fitted into a multivariable logistic regression model. Statistical significance was declared at a two‐sided *p*‐value less than 0.05.

**Results:**

Of 834 sampled individuals, 794 completed the study (95.2% response rate). The PIH prevalence was 5.2% (95% CI: 3.7%, 7.0%). Multivariable analysis identified several factors associated with higher odds of PIH: no formal education (AOR = 5.44, 95% CI: 1.47–20.19), multiple pregnancy (AOR = 3.69, 95% CI: 1.07–12.7), family history of chronic hypertension (AOR = 4.04, 95% CI: 1.51–10.8), presence of edema (AOR = 3.84, 95% CI: 1.37–10.7), and alcohol consumption within the past 12 months (AOR = 6.19, 95% CI: 1.11–34.4). Conversely, receiving nutritional counseling during ANC was associated with reduced odds of developing PIH (AOR = 0.27, 95% CI: 0.09–0.79).

**Conclusions:**

PIH remains a notable health concern among pregnant women in Asella town, showing prevalence comparable to national averages. The identified factors emphasize the interplay between non‐modifiable traits and modifiable behaviors. Strengthening routine ANC services through standardized nutritional counseling may serve as an effective, accessible preventive approach in low‐resource primary care settings.

**Abbreviations**
ANC: antenatal care
AOR: adjusted odds ratio
BP: blood pressure
CI: confidence interval
COR: crude odds ratio
CVD: cardiovascular disease
DBP: diastolic blood pressure
EDHS: Ethiopia Demographic and Health Survey
OR: odds ratio
PIH: pregnancy‐induced hypertension
SBP: systolic blood pressure
SD: standard deviation
STROBE: Strengthening the Reporting of Observational Studies in Epidemiology
WHO: World Health Organization

## 1 Introduction

### 1.1 Background/Rationale

Complications unique to women that develop during pregnancy can impede a healthy gestation; therefore, healthcare professionals are the primary drivers of safety by averting these barriers. Pregnancy‐induced hypertension (PIH) is defined as hypertension that occurs after 20 weeks of gestation in women with previously normal blood pressure. If unmanaged, both the mother and fetus are at significant risk, demanding careful monitoring and expert care.

PIH is a major challenge in antenatal practice due to its burden on obstetric and fetal outcomes. Hypertension plays a significant role in up to 14% of complications during pregnancy and the postpartum period [1]. Hypertensive disorders of pregnancy encompass pre‐existing (chronic) hypertension, gestational hypertension, preeclampsia, and eclampsia, with an estimated global prevalence of 5% to 10% among women of reproductive age [2, 3]. These disorders are significant contributors to maternal and perinatal mortality, accounting for 343,000 maternal deaths annually [1] and 10% to 15% of maternal deaths in low‐ and middle‐income countries [4, 5].

A multisite study in India, Mozambique, Nigeria, and Pakistan found that one out of 10 pregnant women had hypertension [6]. PIH is also a significant risk factor for developing gestational diabetes mellitus [7]. Furthermore, the vulnerability to cardiovascular diseases (CVD) later in life is higher among women with a history of PIH [8, 9, 10], likely due to shared risk factors such as type 2 diabetes, chronic hypertension, and raised blood lipids associated with rapid urbanization and changing lifestyles [11, 12].

The incidence of PIH is rising and is linked to fetal growth retardation and undesirable birth outcomes [13]. Annually, approximately 287,000 women die from preventable causes related to childbirth; 14% of these deaths are linked to PIH, and 61% occur in sub‐Saharan Africa [1, 14, 15]. The magnitude of PIH ranges from 1.8% in the Middle East to 4.5% in the Americas [16], but it is estimated to affect one in 10 women in Africa [17].

In Ethiopia, studies report that up to 10% of maternal mortality is caused by PIH [18]. Despite the routine monitoring of blood pressure as a standard part of antenatal care (ANC), the condition remains a critical challenge. While established risk factors include maternal age, marital status, education, parity, gravidity, gestational age, and history of co‐morbidities [19, 20, 21, 22, 23], national pooled averages often obscure significant regional disparities driven by localized structural, cultural, and behavioral variations. Prior Ethiopian research has focused primarily on northern and southwestern urban clinical tertiary centers, leaving a clear geographic gap in documentation regarding the expanding peri‐urban populations of Southeast Ethiopia. Unique local factors—such as regional variations in alcohol profiles, distinct nutritional patterns, and highly variable accessibility to comprehensive ANC counseling—mean that centralized national data cannot be uniformly applied to local contexts. Consequently, mapping the true disease burden and unique risk configurations within Asella Town is crucial for moving beyond broad national estimates and designing targeted, effective health policies and maternal safety programs. To address this critical gap, this study aims to assess the prevalence of PIH and identify its associated risk factors among pregnant women attending antenatal care at public health facilities in Asella Town, Southeast Ethiopia.

## 2 Methods

### 2.1 Study Design

A facility‐based cross‐sectional study was conducted among pregnant women attending antenatal care (ANC) services to identify determinants associated with pregnancy‐induced hypertension.

### 2.2 Setting

The study was conducted from December 1 to 30, 2023, in Asella Town, located in the Arsi Zone of Oromia Regional State. The town is situated 175 km southeast of Addis Ababa. Healthcare services in the area are provided through three public facilities: Asella Referral and Teaching Hospital, Asella Health Center, and Halila Health Center. Collectively, these facilities serve an average of 2386 pregnant women attending ANC services per month [24].

### 2.3 Participants

The study population comprised randomly chosen pregnant women who were attending ANC at the aforementioned public health facilities during the study period. Eligible participants included pregnant women at a gestational age greater than 20 weeks who were attending routine ANC appointments during the December 2023 study window. To ensure maternal ethical protection and legal status for independent consent, women under 18 years of age, or those suffering from critical communication impairments, were excluded.

### 2.4 Variables

□ **Outcome Variable:** Pregnancy‐Induced Hypertension (PIH), categorized as a binary outcome (Yes/No).

Independent variables were grouped into three primary conceptual domains: **(1) Socio‐demographic and structural factors**, which included age, residence, family size, religion, ethnicity, education, occupation, distance to health facility, and monthly family income; **(2) Obstetric and clinical factors**, comprising age at first pregnancy, birth spacing, ANC visits, gravidity, gestational age, multiple pregnancy, history of PIH, family history of PIH, family history of chronic hypertension, diabetes‐related history, kidney disease, cardiac disease, and presence of edema; and **(3) Behavioral and ANC‐related factors**, encompassing nutritional advice, alcohol consumption during pregnancy, alcohol consumption during the past 12 months, coffee consumption, physical exercise, traditional treatment, tobacco use, exposure to household smoking, khat chewing, fruit consumption, and psychological stress.

### 2.5 Data Sources and Measurement

Data were collected using a structured, interviewer‐administered questionnaire adapted from the Ethiopian Demographic and Health Survey and relevant literature. Blood pressure was measured twice, 6 h apart, by trained health professionals using a standard mercury sphygmomanometer. PIH was defined as an elevated blood pressure reading BP ≥ 140/90 mmHg taken twice at least 6 h apart in a previously normotensive woman after 20 weeks of gestation.

### 2.6 Bias

To minimize selection bias, participants were selected using a systematic random sampling technique. Measurement bias was mitigated through daily calibration of blood pressure apparatuses, standardized measurement protocols, and pre‐testing the data collection tool on 5% of the sample in a non‐study health facility prior to actual data collection. To reduce recall bias, interviewers utilized local event calendars and landmark reference points to assist participants in accurately recalling clinical and obstetric events.

### 2.7 Study Size

The required sample size calculation was based on the double population proportion formula utilizing EPI INFO version 7. Based on gravidity as a primary determinant factor (P1 = 40.9%, P2 = 51.3%, OR = 1.52) [25, 26], and configured with an alpha level of 0.05, 80% power, and an estimated non‐response buffer of 10%, leading to an optimal cohort size of 834 participants.

To select these participants, systematic random sampling was deployed. The total monthly operational volume across the three targeted public facilities (Asella Referral and Teaching Hospital, Asella Health Center, and Halila Health Center) was identified as *N* = 2386 from the previous year's health office audit. The sampling interval was calculated as:

k=N/n=2386/834∼3

Every third pregnant woman who registered for care was systematically approached for recruitment. The index participant for each clinic session was selected utilizing a computer‐generated random number between 1 and 3.

### 2.8 Quantitative Variables

Quantitative variables like age and income were summarized as means and further categorized into ordinal tiers for regression analysis. Blood pressure readings were treated as continuous for descriptive purposes but transformed into a categorical outcome (PIH vs. Normotensive) for the multivariable model.

### 2.9 Statistical Analysis

Data were cleaned in EpiData version 4.6.0.1 and exported to Stata version 17.0 for analysis. Descriptive statistics were computed for all baseline characteristics. Bivariable logistic regression was performed to calculate crude odds ratios (CORs). Candidate variables with *p* < 0.25 in bivariable screening were included in the multivariable logistic regression model to adjust for confounding. Statistical significance was declared at a two‐sided *p* < 0.05. Model fitness was verified using the Hosmer–Lemeshow goodness‐of‐fit test, where *p* > 0.05 demonstrated adequate model fit.

## 3 Results

### 3.1 Socio‐Demographic and Structural Characteristics

A total of 794 pregnant women participated in the study. Nearly half of the respondents (387/794, 48.7%) were between 27 and 36 years of age, with an overall mean age of 27.4 ± 5.2 years. The vast majority were married (748/794, 94.2%), resided in urban areas (569/794, 71.7%), and belonged to the Oromo ethnic group (561/794, 70.7%). In terms of religious affiliation, 47.6% (378/794) were Orthodox Christian, 38.7% (307/794) were Muslim, and 13.5% (107/794) were Protestant. Regarding formal education, 40.3% (320/794) had attained a diploma or above, whereas 7.2% (57/794) had no formal education. Nearly half of the participants were housewives (388/794, 48.9%), followed by government employees (194/794, 24.4%). The mean family size was 3.59 ± 1.49 members, while the median monthly family income was 4000 ETB (IQR = 2000–6000). The median estimated travel time to the nearest health facility was 30.0 min (IQR = 20.0–45.0) (Table 1).

**Table 1:** Baseline characteristics of pregnant women attending antenatal care in Asella Town, Ethiopia, 2023 (*N* = 794).

Domain and variable	Category	Frequency (*n*)	Percentage (%)
Socio‐demographic and structural characteristics			
Age (years)	18–26	321	40.4
	27–36	387	48.7
	≥ 37	86	10.9
	*Mean ± SD*		27.4 ± 5.2
Marital status	Married	748	94.2
	Others (Single/Widowed/Divorced)	46	5.8
Residence	Urban	569	71.7
	Rural	225	28.3
Ethnicity	Oromo	561	70.7
	Others	233	29.3
Religion	Orthodox	378	47.6
	Muslim	307	38.7
	Protestant	107	13.5
	Others	2	0.3
Educational status	No formal education	57	7.2
	Primary school	184	23.2
	Secondary school	233	29.3
	Diploma/Certificate and above	320	40.3
Occupational status	Housewife	388	48.9
	Government employee	194	24.4
	Private sector employee	92	11.6
	Daily laborer	49	6.2
	Student	27	3.4
	Merchant	25	3.1
	Others	19	2.4
Family size	≤ 3 members	415	52.3
	≥ 4 members	379	47.7
	*Mean ± SD (Median)*		3.59 ± 1.49 (3.0)
Average monthly income (ETB)	Median (IQR)		4000 (2000–6000)
Distance to health facility (min)	Median (IQR)		30 (20–45)
Obstetric and clinical characteristics			
Gravidity	Primigravida	226	28.5
	Multigravida	568	71.5
Number of ANC visits	1–2 visits	546	68.8
	≥ 3 visits	248	31.2
History of previous PIH	Yes	8	1.0
	No	786	99.0
Presence of edema	Yes	68	8.6
	No	726	91.4
Behavioral and ANC‐related factors			
Psychological stress	Yes	85	10.7
	No	709	89.3
Nutritional counseling	Yes	685	86.3
	No	109	13.7
Coffee consumption	Yes	682	85.9
	No	112	14.1
Physical exercise	Yes	592	74.6
	No	202	25.4
Current khat chewing	Yes	41	5.2
	No	753	94.8
Current tobacco smoking	Yes	11	1.4
	No	783	98.6

### 3.2 Obstetric, Clinical, and Behavioral Determinants

Obstetrically, most women were multigravida (568/794, 71.5%), and 68.8% (546/794) had attended two or fewer antenatal care (ANC) visits for their current pregnancy. A documented history of previous pregnancy‐induced hypertension (PIH) was recorded in 1.0% (8/794) of participants, while physical edema was detected in 8.6% (68/794). Regarding behavioral and ANC‐related factors, 10.7% (85/794) of the women reported experiencing psychological stress during pregnancy. Most participants received nutritional counseling during ANC visits (685/794, 86.3%) and reported regular physical exercise (592/794, 74.6%). Daily coffee consumption was reported by 85.9% (682/794) of women, while current substance use included khat chewing in 5.2% (41/794) and active tobacco smoking in 1.4% (11/794) (Table 1).

### 3.3 Outcome Data—Prevalence of Pregnancy‐Induced Hypertension

**Figure 1:**
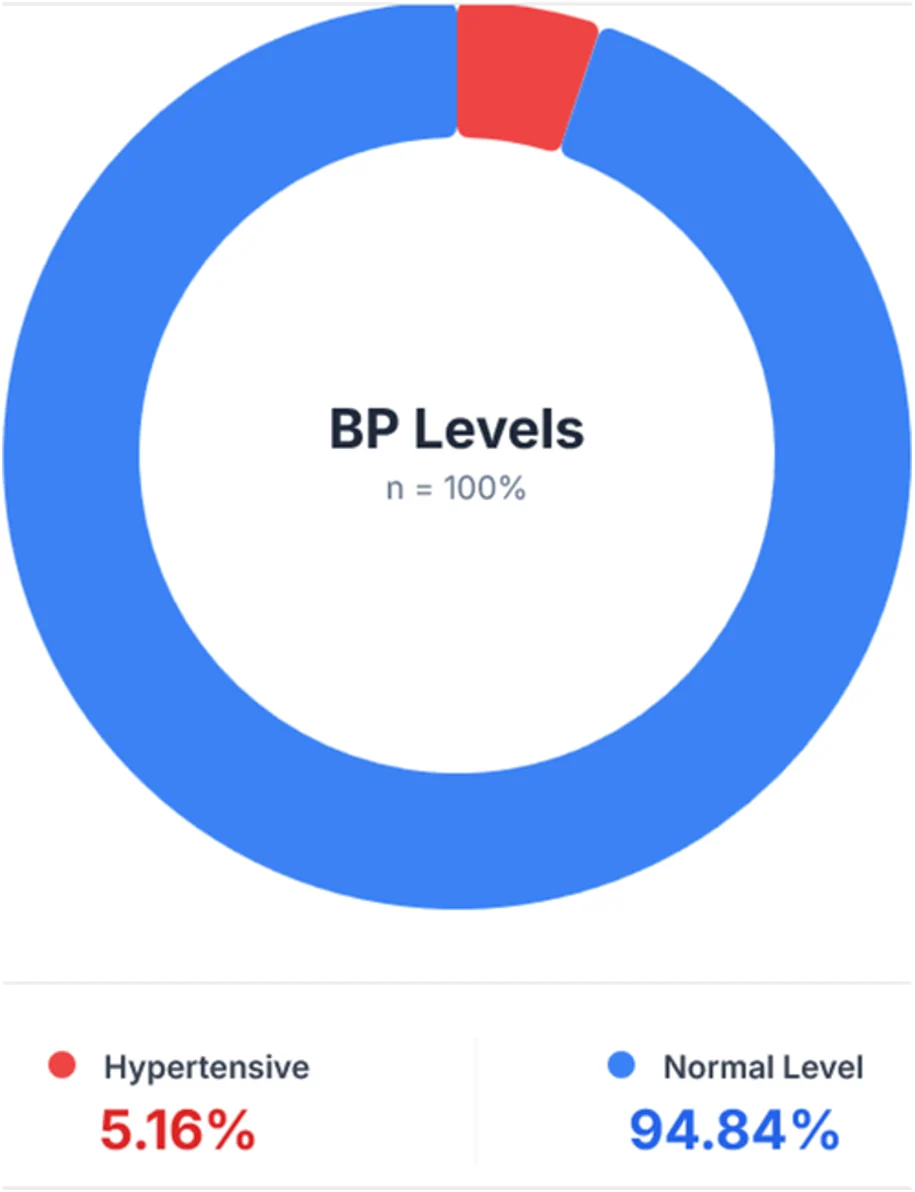
Prevalence of pregnancy‐induced hypertension among study participants, Asella Town, 2023 (*n* = 794).

Among the active analytical sample (*n* = 794), exactly 41 individuals met the criteria for PIH, yielding an overall prevalence of 5.2% (95% CI: 3.7%, 7.0%). Across the entire sample, the mean systolic blood pressure (MSP) was 109 ± 12.45 mmHg, and the mean diastolic blood pressure (DBP) was 70 ± 9.85 mmHg (Figure 1).

### 3.4 Main Results―Multivariable Analysis of Associated Factors

In the multivariable logistic regression model, six independent variables were identified as statistically significant determinants of PIH (Table 2):

**Table 2:** Factors independently associated with pregnancy‐induced hypertension among pregnant women attending ANC in Asella Town, Ethiopia, 2023 (*N* = 794).

Factor	AOR (95% CI)	*p*‐value
No formal education (vs. certificate and above)	5.44 (1.47, 20.19)	0.011
Multiple pregnancy (Yes vs. No)	3.69 (1.07, 12.7)	0.038
Family history of chronic hypertension (Yes vs. No)	4.04 (1.51, 10.8)	0.006
Presence of edema (Yes vs. No)	3.84 (1.37, 10.7)	0.011
Alcohol intake in past 12 months (Yes vs. No)	6.19 (1.11, 34.4)	0.037
Nutritional advice during ANC (Yes vs. No)	0.27 (0.09, 0.79)	0.018

**Detailed Findings on Associated Factors:**

- **Educational Status:** Women with no formal education were 5.44 times more likely to develop PIH compared to those with higher education (AOR = 5.44, 95% CI: 1.47–20.19).
- **Family History:** A positive family history of chronic hypertension increased the odds of PIH by 4.04 times (AOR = 4.04, 95% CI: 1.51–10.8).
- **Multiple Pregnancy:** Women with multiple pregnancies were 3.69 times more likely to have PIH (AOR = 3.69, 95% CI: 1.07–12.7).
- **Presence of Edema:** The clinical observation of edema was associated with a 3.84‐fold increase in the likelihood of PIH (AOR = 3.84, 95% CI: 1.37–10.7).
- **Alcohol Consumption:** Drinking alcohol in the past 12 months was a strong predictor, increasing the risk by 6.19 times (AOR = 6.19, 95% CI: 1.11–34.4).
- **Nutritional Advice:** Receiving dietary and nutritional counseling during antenatal care (ANC) served as a protective factor, associated with a 73% reduction in the odds of PIH (AOR = 0.27, 95% CI: 0.09–0.79).

## 4 Discussion

### 4.1 Key Results

This study revealed that the prevalence of PIH among pregnant women attending antenatal care in Asella Town was 5.2%. The multivariable logistic regression identified no formal education, family history of chronic hypertension, multiple pregnancy, clinical edema, and alcohol consumption as significant independent risk factors for PIH. Conversely, receiving routine nutritional counseling during ANC served as a substantial protective factor against the development of PIH.

#### 4.1.1 Interpretation

The observed PIH prevalence of 5.2% aligns closely with the pooled national prevalence in Ethiopia (6.82%) as well as facility‐based findings from Tikur Anbessa Specialized Hospital (6.69%) and Jimma University Specialized Hospital (5.3%) [18, 25, 27]. However, this figure is notably lower than estimates reported in Arba Minch (18.3%) and Mettu Karl Hospital (12.4%) [28, 29]. These regional variations may stem from differences in study periods, local dietary and lifestyle habits, or the effective implementation of focused ANC services in Asella Town, which facilitates earlier detection and preventive management.

Pregnant women with no formal education exhibited over five‐fold higher odds of developing PIH (AOR = 5.44), consistent with evidence from Jijiga [26]. Lower educational attainment often correlates with reduced health literacy regarding hypertensive warning signs, suboptimal health‐seeking behavior, and reduced engagement with preventive prenatal care. Furthermore, a positive family history of chronic hypertension quadrupled the odds of PIH (AOR = 4.04), echoing trends reported in Gondar and Jimma [25, 30]. This underscores an underlying genetic predisposition to vascular hyper‐reactivity and endothelial dysfunction that becomes unmasked under the physiological strain of pregnancy.

Women with multiple pregnancies were 3.69 times more likely to develop PIH compared to those carrying singletons (AOR = 3.69). Mechanistically, multiple gestations induce excess placental mass, triggering an overproduction and systemic release of antiangiogenic factors—such as soluble fms‐like tyrosine kinase‐1 (sFlt‐1)—into the maternal circulation. These factors antagonize vascular endothelial growth factor (VEGF) and placental growth factor (PlGF), promoting widespread endothelial injury and systemic hypertension [31].

The clinical detection of peripheral edema was also strongly associated with PIH (AOR = 3.84). While physiological edema is common in late gestation, pronounced fluid retention often reflects impaired renal sodium handling and increased capillary permeability secondary to systemic endothelial activation in hypertensive disorders.

The strong association between alcohol intake in the preceding 12 months and PIH (AOR = 6.19) is particularly notable and concurs with observations in Arba Minch and Gondar [28, 30]. Alcohol acts as a potent modifiable cardiovascular toxicant, promoting vascular oxidative stress, activating the renin‐angiotensin‐aldosterone system (RAAS), and impairing endothelial nitric oxide production.

Conversely, nutritional counseling during ANC demonstrated a robust protective effect, associated with a 73% reduction in the odds of PIH (AOR = 0.27). Standardized dietary guidance during prenatal visits equips mothers to manage gestational weight gain, reduce dietary sodium intake, and maintain adequate micronutrient supplementation, thereby mitigating physiological triggers of vascular resistance.

### 4.2 Limitations

The cross‐sectional design of this study inherently limits our ability to establish temporal or definitive causal relationships between the identified risk factors and the onset of PIH. Additionally, self‐reported behavioral variables—such as pre‐pregnancy alcohol consumption—may be susceptible to social desirability and recall biases. Finally, because data collection was conducted exclusively within public health facilities, these findings may not fully capture the socio‐demographic or clinical profile of pregnant women utilizing private healthcare services.

### 4.3 Generalizability

The findings are broadly generalizable to urban and peri‐urban maternal populations in Central and South‐eastern Ethiopia where prenatal services are primarily accessed through public healthcare systems. The rigorous sampling strategy, high response rate (95%), and robust analytical sample size (*N* = 794) contribute to high internal and external validity within similar low‐resource settings.

## 5 Conclusion and Recommendations

### 5.1 Conclusion

The prevalence of PIH in Asella Town (5.2%) is comparable to national estimates. The disease burden is driven by an interplay of unmodifiable genetic factors (family history), obstetric strain (multiple gestations), clinical markers (edema), and modifiable behavioral choices (alcohol consumption and lower educational exposure). Structured nutritional counseling during prenatal care represents the most effective non‐pharmacological protective intervention in this setting.

### 5.2 Recommendations

- **For Health Facilities and ANC Providers:** Standardize and enforce structured nutritional counseling at every prenatal contact. Protocolize targeted risk stratification and frequent blood pressure monitoring for high‐risk cohorts—specifically women with multiple gestations or a family history of hypertension.
- **For Local Health Bureaus:** Launch targeted, community‐level maternal health campaigns addressing the risks of alcohol intake prior to and during pregnancy, with tailored outreach for women with lower formal education.
- **For Researchers:** Conduct prospective cohort studies starting in early pregnancy to establish the precise temporal sequence of these risk factors and evaluate the longitudinal impact of early nutritional interventions on maternal and perinatal outcomes.

## Author Contributions

**Birtukan Ahmed:** conceptualization, methodology, formal analysis, investigation, data curation, writing – original draft, and project administration. **Ayalneh Demissie:** methodology, validation, investigation, formal analysis, writing – review and editing, supervision, and final approval of the version to be published. **Biniam Belete:** software, validation, and visualization. **Addis Wordofa:** resources, funding acquisition, writing – review and editing. **Helen Bekele:** data curation, validation, and visualization. **Tesfa Gebremeskel:** methodology, software, validation, and writing – review and editing. All authors have read and agreed to the published version of the manuscript.

## Funding

The authors have nothing to report.

## Ethics Statement

The study was conducted in strict accordance with the ethical tenets of the Declaration of Helsinki. Formal ethical approval was granted by the Institutional Review Board (IRB) of Rift Valley University College, Department of Public Health, Adama Campus, under protocol code RVU/ERC/61/2023 on October 9, 2023, as documented on the official Ethical Review Committee Approval Sheet. All authors have read and approved the final version of the manuscript. Dr. Ayalneh Demissie had full access to all of the data in this study and takes complete responsibility for the integrity of the data and the accuracy of the data analysis.

## Consent

Administrative permission was obtained from the medical directors of each participating health facility. Because all participants were verified adults aged 18 years or older, direct informed consent was obtained from each subject prior to enrollment. Participants were given an explicit briefing regarding the study's scope, their right to immediate voluntary withdrawal without clinical penalty, and our rigorous data anonymization protocols. Written or clear thumbprint informed consent was secured from every participant, and all data records were completely de‐identified.

## Conflicts of Interest

The authors declare no conflicts of interest.

## Transparency Statement

Dr. Ayalneh Demissie affirms that this manuscript is an honest, accurate, and transparent account of the study being reported; that no important aspects of the study have been omitted; and that any discrepancies from the study as planned have been fully explained.

## Supporting information


Supporting File

## Data Availability

The authors confirm that the data supporting the findings of this study are available within the article and its supporting materials (“PIH_data_anonymized.csv”).
